# Tuning coercive force by adjusting electric potential in solution processed Co/Pt(111) and the mechanism involved

**DOI:** 10.1038/srep43700

**Published:** 2017-03-03

**Authors:** Cheng-Hsun-Tony Chang, Wei-Hsu Kuo, Yu-Chieh Chang, Jyh-Shen Tsay, Shueh-Lin Yau

**Affiliations:** 1 Department of Physics, National Taiwan Normal University, Taipei 116, Taiwan; 2 Department of Chemistry, National Central University, Jhongli 320, Taiwan

## Abstract

A combination of a solution process and the control of the electric potential for magnetism represents a new approach to operating spintronic devices with a highly controlled efficiency and lower power consumption with reduced production cost. As a paradigmatic example, we investigated Co/Pt(111) in the Bloch-wall regime. The depression in coercive force was detected by applying a negative electric potential in an electrolytic solution. The reversible control of coercive force by varying the electric potential within few hundred millivolts is demonstrated. By changing the electric potential in ferromagnetic layers with smaller thicknesses, the efficiency for controlling the tunable coercive force becomes higher. Assuming that the pinning domains are independent of the applied electric potential, an electric potential tuning-magnetic anisotropy energy model was derived and provided insights into our knowledge of the relation between the electric potential tuning coercive force and the thickness of the ferromagnetic layer. Based on the fact that the coercive force can be tuned by changing the electric potential using a solution process, we developed a novel concept of electric-potential-tuned magnetic recording, resulting in a stable recording media with a high degree of writing ability.

During the past decade, data storage has become one of the most pressing issues that has confronted our society and the information age. The increasing use of the cloud for the storage of digital information involves the use of hard disk drives which use magnetism for the storage and retrieval of digital information^1^. In 2014, about 0.56 billion hard disk drives that provided more than 540 exabytes of storage capacity were produced^1^,^2^. However, due to problems associated with increasing the storage capacity of hard disk drives, the data storage community faces the necessity of exploring and developing new concepts in attempts to increase areal density^1^,^2^,^3^. In recent years, extensive efforts have been devoted to exploring alternate approaches for improving the storage density of magnetic recording devices, and include two-dimensional magnetic recording (TDMR)^4^,^5^, heat-assisted magnetic recording (HAMR)^6^,^7^,^8^,^9^, microwave-assisted magnetic recording (MAMR)^10^,^11^, bit-patterned magnetic recording (BPMR)^12^,^13^,^14^,^15^, and optical switching of magnetic domains^16^,^17^,^18^. For instance, a broadband near-field thermal extraction device based on hyperbolic metamaterials can significantly enhance near-field energy transfer by extracting evanescent waves with arbitrarily large lateral wave vectors^6^. Near-field thermal extraction has important practical implications in HAMR^6^,^8^. Free-floating magnetic structures composed of *in situ* cross-linked magnetically assembled nanoparticles have been reported to retain their patterned shape during manipulation with external magnetic fields^12^ while a magnetic pattern can serve as an elementary base in magnetic recording media such as BPMR^12^,^14^. By exploiting the field-confining capability as plasmonic nanoantennas, ferrimagnetic TbFeCo thin films have the potential for use in the optical switching of magnetic domains, while the importance of a chemically homogeneous sample structure is highlighted for optical switching-based recording technologies^16^. The diazotization of Ru(bpy)_2_(phen-NH_2_)^2+^ in the presence of carbon nanotubes (CNT) results in the formation of nanodots on CNTs, which are capable of transducing photo stimuli into electricity and magnetism at ambient conditions, and the resultant photomagnetic CNTs are a multifunctional material that have the potential for use in in optical switching-based recording technologies^17^.

Because of the higher storage density^19^,^20^,^21^ and lower power consumption^22^,^23^,^24^,^25^, the electric potential controlled magnetic device has attracted interest in the area of magnetic recording research. Exploring electric potential tuned magnetic devices in electrolytic conditions has also attracted great interest^24^,^25^,^26^,^27^,^28^ because the solution process is a low cost, rapid method that is amenable for use in commercial production. In the first report of electric potential induced magnetism using FePt and FePd films in NaOH as an electrolyte, the coercive force (*H*_C_) could be enhanced by about 4.5% by varying the electric potential between −1000 and −400 mV^26^. This pioneering work has motivated researchers to further examine electric potential tuned magnetism in electrolytes^24^,^25^,^27^,^28^. For example, MgO(2.0 nm)/Co (0.4 nm)/Pt (1.2 nm)/Ta (3.0 nm) films show magnetic switching near the coercive field when the electric potential is tuned from +2000 to −2000 mV in an ionic liquid electrolyte^24^. The ferromagnetic proximity effect shows that an electric field can be used to control the magnetic moments in films made of Pd, originally a nonmagnetic element^25^. An electrochemical etching process can be used to reduce the thickness of Co films with less surface defects while the electric field induced depression of the *H*_C_ was observed to be reproducible for the case of an electrochemically etched specimen^28^. The focus of this research was on electric potential tuned magnetic phenomena that occur via the solution process with the characteristics of highly efficient controlling and ease of production.

Exploring the possible mechanisms responsible for developing electric potential tuned magnetic phenomena is an important issue in terms of developing more efficient magnetic recording devices. From the literature, the mechanisms of the electric potential tuned magnetic phenomena are mainly related to the Curie temperature^24^,^27^,^29^ and magnetic anisotropy energy (MAE)^30^,^31^,^32^,^33^. For example, the Curie temperature of Ga_0.93_Mn_0.07_As increases as the gate voltage sweeps from positive to negative values^29^. Based on first principle calculations of the electric field effects on magnetic anisotropy for ferromagnetic thin films on Pt(111), the MAE increases with increasing electric field and decreases with decreasing electric field^30^. Giant electric-field-assisted modifications of the interfacial magnetic anisotropy have been reported for the case of a half-metallic Heusler compound/MgO interface^31^. However, the mechanism responsible for how different thicknesses of the ferromagnetic layer (*t*_FM_) affect electric potential tuned magnetic phenomena is still unclear.

In this letter, tuning the magnetic properties by an electric potential for Co/Pt(111) of nanometer thickness in the Bloch-wall regime was investigated for a solution process. For Co/Pt(111) in the Bloch-wall regime, the depression of the *H*_C_ is detected by applying a negative electric potential in an electrolytic condition. The electric potential tuned *H*_C_ and *t*_FM_ shows an inverse square relation that can be successfully explained using an electric potential tuning-magnetic anisotropy energy (EPT-MAE) model. For a smaller *t*_FM_, the electric potential induced change of the *H*_C_ is larger, while the electric potential induced change of the MAE is again magnified. A higher controlling efficiency of the tunable *H*_C_ in thinner ferromagnetic layers was demonstrated using our apparatus. Based on the characteristics of a tunable *H*_C_ produced by changing the electric potential using a solution process, we propose a novel technique for achieving electric-potential-tuned magnetic recording (EPTMR). A schematic plot of the EPTMR with storage and writing processes is shown in Fig. 1. For a storage pillar, the H_C_ is tunable by a negative electric potential, and ranged from a storage area of high H_C_ to a writing area of relatively small H_C_ within an available head field range. In additional, the patterned pillars are isolated in write or read process because of the parallel potential channel design. The parallel potential channel is designed to switch the write and read process by changing the supplied electric potential for each pillar that can avoid the exchange coupling between pillars. In these design, the potential controllable magnetic pillars can be structured as three-dimensional stacking like many electronic devices^34^ which improve the amount of pack clusters and enhance the areal density in a disc. Combining the solution process and controlling the electric potential for the operation of a magnetic device is a viable technique for achieving a highly controlled efficiency and a lower power consumption with a reduced cost of production. The developed EPTMR shows promise for use in applications in which the solution process is combined with electric potential control for magnetic data storage.

After waiting for a certain time at a suitable potential for Co deposition, a linear potential scan in the positive potential direction is performed to strip off the Co from the film. Anodic peaks from cyclic voltammograms (CV) for strip off measurements for Co/Pt(111) thinner than 18 nm are shown in Fig. 2a. The thickness of the cobalt can be obtained from the strip off method by measuring the quantity of electric charge, by integrating the charge under the anodic peak^28^,^35^. Figure 2b shows Kerr signals versus the magnetic field measured at electric potential *V* = −500 mV for a Co/Pt(111) layer thinner than 18 nm. No hysteresis is detected in the polar configuration. Hysteresis occurs only in the longitudinal configuration. This shows that the easy axis of magnetization lies on the surface plane. Shape anisotropy is dominant for the Co/Pt(111) in this thickness range^36^,^37^.

Figure 3a shows the saturated Kerr intensities in both the longitudinal and polar configurations versus the Co thickness (*t*_Co_). As the *t*_Co_ increases, the saturated Kerr intensity in the longitudinal configuration increases between 4.5 and 18 nm, due to the relation between the magnetization and the thickness of the ferromagnetic layers^38^. This phenomenon has also been reported for Co/Cu(001) films in electrolytic conditions^39^ and for Mn-doped ZnO films^40^. The relation between the saturation magnetization (*M*_S_) and *t*_FM_ can be expressed by


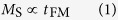


by considering the amount of magnetic moments in a ferromagnetic layer^38^,^39^,^40^,^41^,^42^. A plot of the coercive force of Co/Pt(111) versus *t*_Co_ is shown in Fig. 3b. The value for *H*_C_ increases rapidly with increasing *t*_Co_ below 6.8 nm. From the literature, Bloch wall energy density decreases with increasing film thickness because of the increased magnetostatic energy due to the appearance of charged surface above and below the wall, while the Néel wall energy shows opposite trend because of the proportional relation with the area of the charged surface inside the film^43^. The analysis of the demagnetization energy for Néel-wall regime shows that the increase of the *H*_C_ by increasing the film thickness^44^,^45^. As the thicknesses of magnetic layers increase, the change from of domain-wall types from Néel wall to Bloch wall has been reported for different magnetic materials in nanometer thicknesses^43^,^44^,^45^,^46^,^47^. For Co/Pt(111) films thicker than 6.8 nm, *H*_C_ decreases monotonously which follows Bloch wall behavior^35^,^44^,^48^. By increasing *t*_FM_, the increase in *H*_C_ to a maximum at around several nanometers followed by a monotonous decrease for thicker films has been previously reported and can be described by changing the domain wall type from a Néel wall to a Bloch wall^43^.

In order to elucidate the role of the electric potential on the magnetic properties of Co/Pt(111) in Néel-wall and in Bloch-wall regimes, Fig. 4a and b show Kerr signals versus the magnetic field for 4.5 and 6.8 nmCo/Pt(111), respectively. For Co/Pt(111) in the Néel-wall regime (*t*_Co_ = 4.5 nm), no significant change in *H*_C_ is detected at different electric potentials. The Néel-wall is related to the film thickness in order to minimize the magnetostatic energy. At a sufficiently small film thickness, the magnetostatic energy is no longer significant and the Néel-wall is stabilized. In addition, the energy density of the Néel-wall is related to its magnetization and film thickness^43^. Therefore, the electric potential might not easily induce a change in the Néel-wall in few hundred millivolts. For Co/Pt(111) in the Bloch-wall regime (*t*_Co_ = 6.8 nm), an electric potential induced change in *H*_C_ occurs. For a larger negative electric potential (*V* = −500 mV), the value for *H*_C_ is smaller. This result provides strong evidence for the negative electric potential induced depression in *H*_C_ for Co/Pt(111) in the Bloch-wall regime. Variations in *H*_C_ are shown in Fig. 4c for Co/Pt(111) in the Bloch-wall regime under conditions where the electric potential is repeatedly changed between *V* = −500 and −400 mV. The jump in *H*_C_ between 0.42 and 0.48 kOe is highly reproducible within hours of the start of the experimental tests. At this electric potential range, the typical current is around few μA/cm^2^. The heating power density by the electric current is less than a few μW/cm^2^and therefore the effects of thermal annealing can be neglected. In additional, in the Bloch-wall regime, the films are robust against variations in the electric potential for magnetic switching^44^. We therefore conclude that controlling *H*_C_ by means of the electric potential for magnetic films in the Bloch-wall regime represents a possible route for preparing spintronic switching devices. In the following discussions for the electric potential induced phenomena, we focus on Co/Pt(111) in the Bloch-wall regime.

As shown in Fig. 5a for Co/Pt(111) with *t*_Co_ between 7 and 18 nm, the *H*_C_ measured at both *V* = −500 and−400 mV decrease monotonously with increasing *t*_Co_. At *V* = −500 mV for an individual *t*_Co_, the reduction in *H*_C_ is more pronounced. By tuning the electric potential *V*, the electric field *E* applied to the specimen can be adjusted. The change in the coercive force (Δ*H*_C_) at electric fields *E*_1_ and *E*_2_ can be defined as


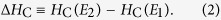


Figure 5b shows plots of Δ*H*_C_ versus *t*_Co_ where the electric fields *E*_1_ and *E*_2_ correspond to the electric potentials *V*_1_ = −500 mV and *V*_2_ = −400 mV, respectively. The value for Δ*H*_C_ decreases with increasing *t*_Co_. In a further analysis of the data in Fig. 5b by taking the logarithms of both the Δ*H*_C_ and *t*_Co_, a well linear relation between ln(Δ*H*_C_) and ln(*t*_Co_) is observed, as shown in Fig. 5c. A slope of −2.00 ± 0.06 was obtained from the linear fitting of the data. This shows that the Δ*H*_C_ is proportional to the inverse of the square of *t*_Co_. Based on the above experimental evidence, we proposed an EPT-MAE model to explain the inverse square relation between the electric potential tuned *H*_C_ and the thickness of the ferromagnetic layer. Detailed discussions are below.

Both the magnetic anisotropy and the imperfections in ferromagnetic materials give rise to hysteresis loss and an increase in the coercive force^44^. Being an upper limit of the *H*_C_, an easy-axis magnetization process results in a square *M-H* loop, and is characterized in the single domain behavior or pinned wall limit by rotational hysteresis, i.e.,


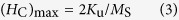


where *K*_u_ is the MAE of the ferromagnetic layer^43^. By considering the hysteresis loss energy for the pinning of the domain wall motion, the lower limit of the coercive force can be expressed by


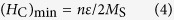


where *n* is the amount of pinning sites and *ε* is the pinning energy for a domain wall motion^44^. Combining Eqs (3) and (4), the coercive force can be expressed as


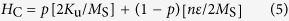


where *p* (0 ≤  *p* ≤ 1) is the percentage of the contribution of the magnetic anisotropy energy. By tuning the electric potential applied to a ferromagnetic film, previous investigations indicate that the corresponding magnetoresistance^49^,^50^, Hall effects^51^,^52^ and magnetization switching^10^,^23^,^28^ are highly reproducible. By choosing suitable electric potential ranges, the changing of pinning sites such as impurities, grain boundary, dislocations, and voids in the ferromagnetic films can be avoided^10^,^23^,^28^,^49^,^50^,^51^,^52^. Assuming that pinning sites are independent of the applied electric potential, the pinning of domain wall motion related to hysteresis loss energy can be neglected for an analysis of Δ*H*_C_. Therefore, from Eq. (5), Δ*H*_C_ can be expressed by


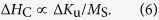


Because the *M*_S_ is proportional to *t*_FM_ as shown in Eq. (1), the relation between the *t*_FM_ and the Δ*H*_C_ can be expressed by


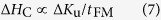


In previous reports, the first principle calculations for Fe and Co films on Pt(111) show that the MAE is enhanced when the electric field *E* is increased^30^. The electric voltage effect on MAE of magnetic layers is expected via charge redistribution among different electron d-orbitals (some or all of d_xy_, d_yz_, d_xz_, d_z_^2^, and d_x_^2^ − _y_^2^) in the presence of electric field^53^. For ultrathin films, it has been reported that the absorption atoms such as H^+^, OH^−^ and CO on the top of Co can influence the interface MAE via controllable electric field effect^54^,^55^. In this report, we start from Co/Pt(111) thicker than 4.5 nm where the MAE contains both the bulk and interfacial contributions. The adsorbed species are H^+^ and K^+^ in the electrolyte while the large electric field created by the electrical double layer near the electrode surface is responsible to the change of the MAE of the magnetic system. The electric field applied on the specimen can be adjusted by tuning the electric potential *V*. The change in the electric field (Δ*E*) can be defined as


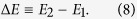


Under different electric fields, the MAEs are different. In this study, the magneto-optical Kerr effect (MOKE) measurements were carried out at electric potentials *V*_1_ = −500 mV and *V*_2_ = −400 mV. By further considering the thickness of the ferromagnetic layer, the relation between the change in magnetic anisotropy energy Δ*K*_u_ at the electric fields *E*_1_ and *E*_2_ (corresponding to *V*_1_ = −500 mV and *V*_2_ = −400 mV, respectively) and the change in the electric field Δ*E* can be expressed as


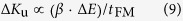


where *β* is the interfacial magnetic anisotropy energy coefficient^31^. By substituting Eq. (9) into Eq. (7), the dependence of Δ*H*_C_ on layer thickness can be expressed as


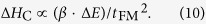


From the discussions of the electric field induced change of MAE, the Δ*H*_C_ versus *t*_FM_ shows an inverse square relation, as shown in Eq. (10). This relation is in good agreement with the experimental data in Fig. 5c where the slope of a plot of ln(Δ*H*_C_) versus ln(*t*_Co_) is −2.00 + 0.06. This result shows that combining MAE and electric field effects can successfully explain the inverse square relation between the Δ*H*_C_ and the *t*_FM_.

Based on Eqs (7, 9, and 10), an EPT-MAE model that explains the relation between the electric potential tuned *H*_C_ and *t*_FM_ is proposed and is schematically illustrated in Fig. 6. For a smaller *t*_FM_, the electric potential induced change in *H*_C_ is larger, as indicated in Eq. (7). The electric potential induced change of MAE again magnifies the change in *H*_C_ as suggested in Eq. (9). The resultant effects are responsible for the inverse square relation between Δ*H*_C_ and *t*_FM_ in Eq. (10). In the left panel of Fig. 6, a more negative electric potential shows the effect of reduction of *H*_C_. Compared to the right panel of Fig. 6 for a half *t*_FM_, the electric potential induced Δ*H*_C_ is enhanced by four times. This shows that the electric potential induced Δ*H*_C_ is much more pronounced for thinner ferromagnetic layers. From a technological point of view, by changing the electric potential in ferromagnetic layers with smaller thicknesses, a higher controlling efficiency of the tunable *H*_C_ is demonstrated in our apparatus. The establishment of the EPT-MAE model provides insights into our knowledge of the relation between electric potential tuning *H*_C_, the ferromagnetic layer thickness, and associated mechanisms.

For magnetic recording applications, superparamagnetic behavior is the limitation for further reducing the volume of a magnetic bit, but EPTMR is a possibility, as shown in Fig. 1. From the literature, in order for magnetic grains in a typical size distribution to remain stable for ~10 years, the ambient temperature stability factor *K*_u_*V/k*_B_*T* needs to be larger than 70 for a commercial device^9^; where *V* is the volume of a nanoparticle; *k*_B_ is the Boltzmann constant; and *T* is the sample temperature. To enhance the areal density of a magnetic recording disc, increasing the MAE is the key point. When using a material with a higher MAE, a higher writing field is necessary, but this is a disadvantage for real applications. By employing an electric potential tuned mechanism, the findings reported here show that 100 mV can reduce the coercive force by 15% for thin magnetic films (6.8 nm Co/Pt(111) as an example) in the Bloch wall regime. In practice, by using EPTMR, it is possible to create stable recording media with a high writing ability.

In conclusion, for Co/Pt(111) of nanometer thickness in the Bloch-wall regime, a depression in *H*_C_ is detected when a negative electric potential is applied in an electrolytic condition. For Co/Pt(111) under conditions of a continuously changing electric potential, the increase in *H*_C_ between two states is highly reproducible. As the value for *t*_FM_ increases, both the *H*_C_ measured at *V* = −500 and −400 mV decrease monotonously. The reduction of *H*_C_ for an individual *t*_Co_ is more pronounced at a more negative potential. An electric potential tuned *H*_C_ and *t*_FM_ have an inverse square relationship with one another. By changing the electric potential in ferromagnetic layers with smaller thicknesses, a higher controlling efficiency of tunable *H*_C_ is achieved. Assuming that the pinning domains are independent of the applied electric potential, an EPT-MAE model in which the MAE and the electric field effects are combined is established. This provides insights into our knowledge of the relation between electric potential tuning *H*_C_ and the thickness of the ferromagnetic layer. The implementing of EPTMR is a potentially promising route for applications combining a solution process and electric potential control in magnetic data storage.

## Methods

A platinum crystal was oriented within 0.5° of the [111] direction as checked by x-ray diffraction. The diameter of the crystal is 8 mm while the thickness is 2 mm. This specimen size is suitable for the designed apparatus for the MOKE measurements in electrochemical environments^28^,^35^,^56^. Before loading into the electrochemical cell, the surface of the crystal was mechanically polished with alumina powder with a diameter down to 0.05 μm and was then annealed using a hydrogen torch. The details of the process for preparing Pt electrode can be found in the literature^28^,^57^,^58^,^59^. All solutions were prepared with high purity chemicals (HCl and CoCl_2_) and ultra-pure water(>18 MΩ cm). To avoid contamination by oxygen, the solutions were purged with high purity argon for 1 hr before the experiments. The electrochemical cell used for the CV and MOKE measurements was developed and built in our laboratory^34^,^56^. Pt and Ag wires were used as the counter electrode and the reference electrode, respectively. The electricity of the standard three-electrode setup was controlled by a potentiostat that was constructed in Bonn^34^,^56^,^60^,^61^. The CV of the Pt(111) electrode in a pure supporting electrolyte containing HCl and KCl, and with a CoCl_2_ additive are shown in the Supplementary Information 1. For the condition of the film deposition, by comparing the CV for specimens in electrolytes with and without CoCl_2_, the electric potential range that is suitable for the electrodeposition of Co/Pt(111) was determined. The Co layer was electrodeposited at an electric potential more negative than −700 mV for a pre-determined time, depending on the thickness of the layer. The potential range for MOKE measurements was chosen to be between −400 and −600 mV to avoid the possible deposition/dissolution of the Co deposits. A He–Ne laser with a wavelength of 632.8 nm was used as the light source for the MOKE measurements. The Kerr signals as a function of magnetic field were measured by a photodiode and recorded by a computer-controlled multimeter to generate the hysteresis loops.

By applying an electric potential, an electrical double layer (EDL) near the electrode surface was established^62^. The thickness of the EDL was in the nanometer scale, thus permitting hundreds of millivolts to create an electric field as large as 10^8 ^V/m^25^,^63^. A schematic plot in Supplementary Information 2 elucidates the specimen structure where the electric field influences the ferromagnetic layer supported on the Pt electrode through the establishment of the EDL. The electric field is responsible for the change in MAE and the related change in the *H*_C_. Surface species are different in different electrolytes, and the effects of point of a zero charge (PZC) need to be taken into consideration. In this report, we used the same electrolyte and the same electrochemical conditions, the PZC was not altered. Discussions of the EDL as well as the PZC can be found in the Supplementary Information 2 and may be of importance for further applications in the case for solution processes.

## Additional Information

**How to cite this article**: Chang, C.-H.-T. *et al*. Tuning coercive force by adjusting electric potential in solution processed Co/Pt(111) and the mechanism involved. *Sci. Rep.* **7**, 43700; doi: 10.1038/srep43700 (2017).

**Publisher's note:** Springer Nature remains neutral with regard to jurisdictional claims in published maps and institutional affiliations.

## Supplementary Material

Supplementary Information

## Figures and Tables

**Figure 1:**
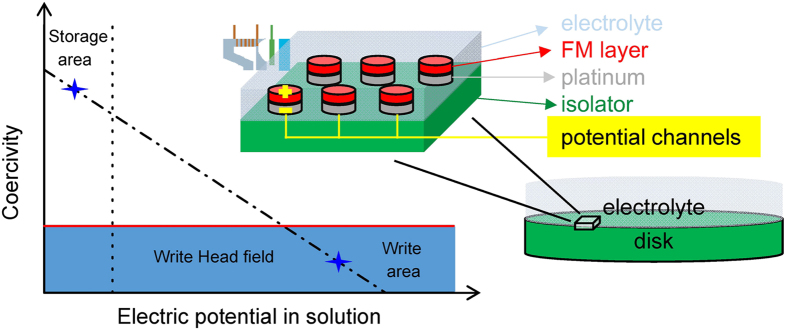
A schematic plot showing the storage and writing processes of EPTMR. The inset shows the designs of a magnetic recording disc for EPTMR.

**Figure 2:**
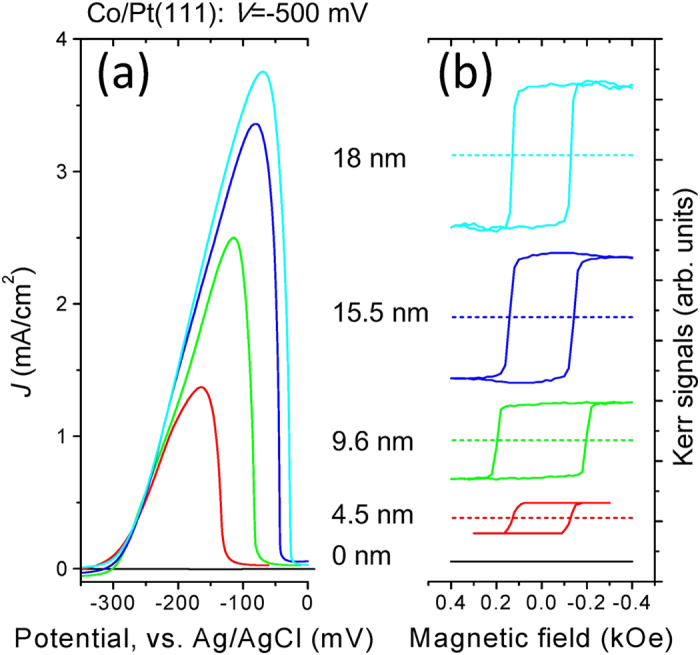
(**a**) The CV of strip off measurements for Co/Pt(111). (**b**) Kerr signals versus the magnetic field measured at *V* = −500 mV for Co/Pt(111) in both the longitudinal (solid lines) and polar (dashed lines) configurations.

**Figure 3:**
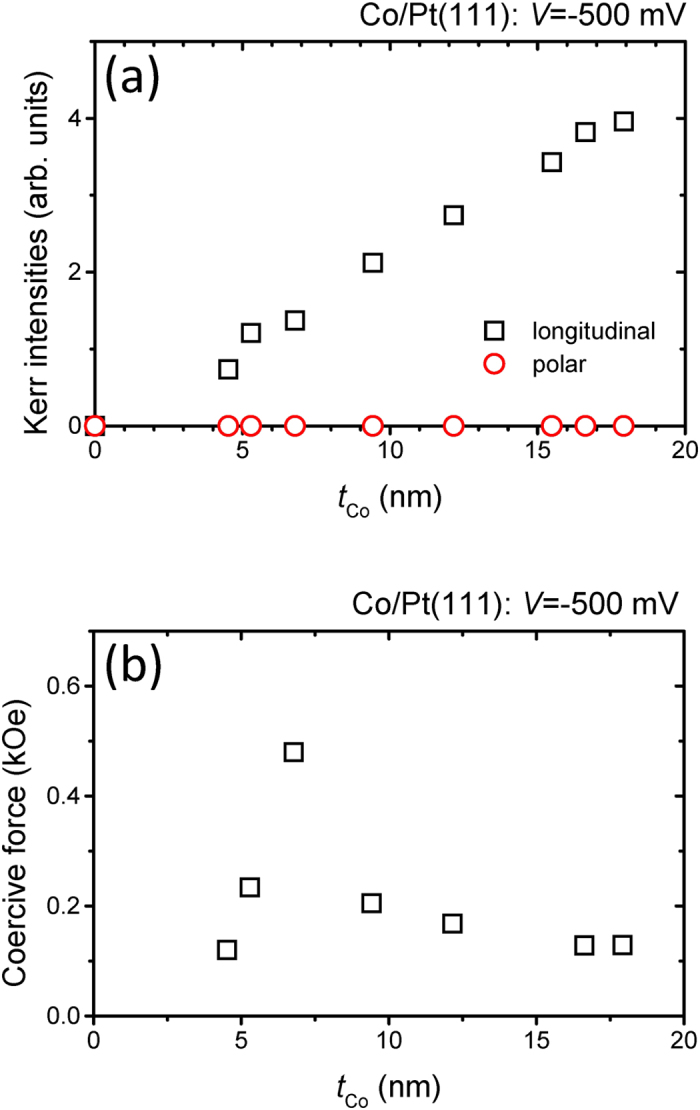
(**a**) Saturated Kerr intensities versus the thickness of the Co layer in both the longitudinal and polar configurations. (**b**) Coercive force versus Co thickness in the longitudinal configuration.

**Figure 4:**
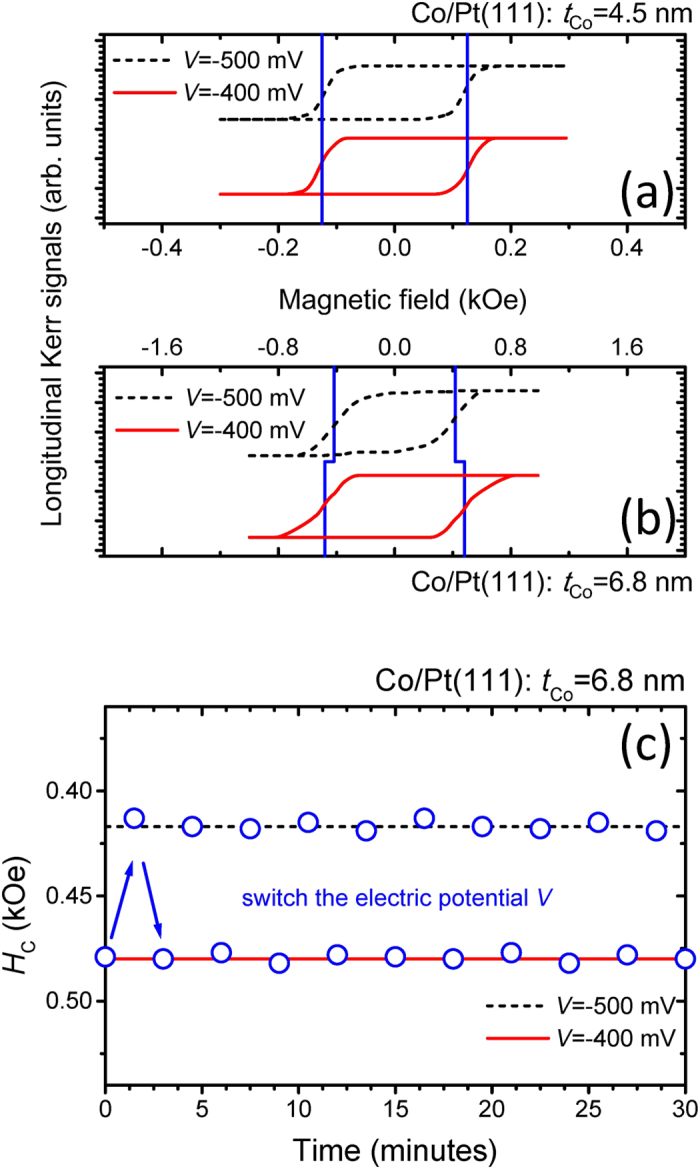
Kerr signals versus the magnetic field measured at *V* = −500 and −400 mV for (**a**) 4.5 and (**b**) 6.8 nm Co/Pt(111) in the longitudinal configuration. (**c**) Variations of the *H*_C_ for 6.8 nm Co/Pt(111) under conditions of continuously changing electric potential *V* = −500 and −400 mV.

**Figure 5:**
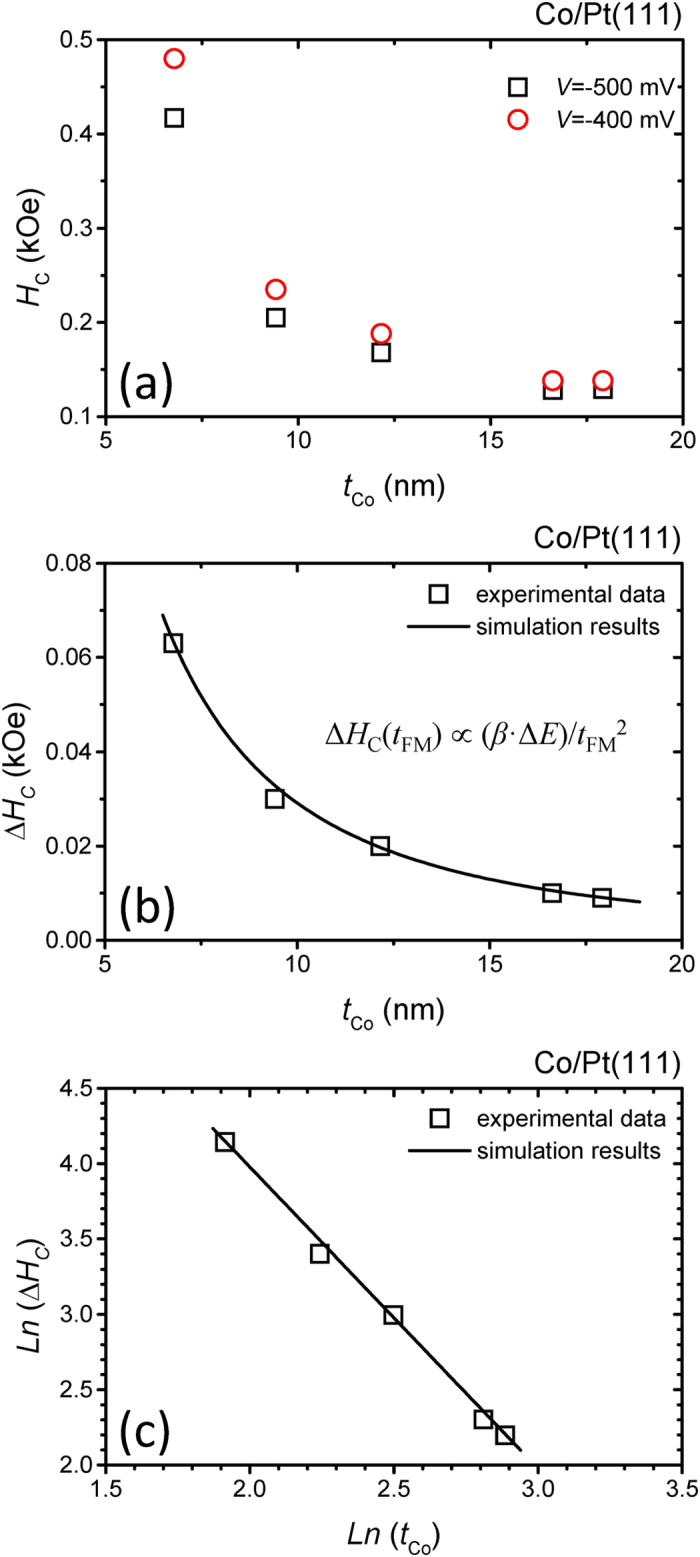
(**a**) *H*_C_ versus Co thickness for Co/Pt(111) with Co thickness thicker than 6.8 nm measured at *V* = −500 and −400 mV in the longitudinal configuration. (**b**) The experimental data and simulation results for Δ*H*_C_ versus the thickness of the Co layer. (**c**) The experimental data and simulation results for the ln(Δ*H*_C_) versus ln(*t*_Co_).

**Figure 6:**
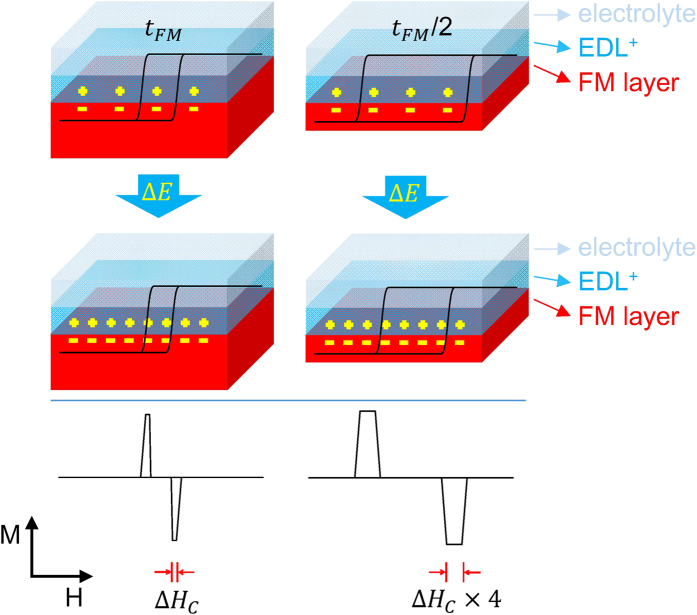
A schematic plot shows the electric potential induced change of the coercive force for ferromagnetic layers with different thicknesses in the Bloch-wall regime.
