# Supplementary material for: Tuning coercive force by adjusting electric potential in solution processed Co/Pt(111) and the mechanism involved
Source: Sci Rep. 2017 Mar 3;7:43700. doi: 10.1038/srep43700 (PMC5334650; doi:10.1038/srep43700)
Supplement: Supplementary Information [file srep43700-s1.pdf]

Supplementary information

Tuning coercive force by adjusting electric potential in solution processed Co/Pt(111) and the mechanism involved

Cheng-Hsun-Tony Chang,[†] *Wei-Hsu Kuo,*[†] *Yu-Chieh Chang,*[†] *Jyh-Shen Tsay,*[*, †] *and Shueh-Lin Yau*[*,§]

[†]Department of Physics, National Taiwan Normal University, Taipei 116, Taiwan

[§]Department of Chemistry, National Central University, Jhongli 320, Taiwan

Supplementary information 1

Ranges of electric potential for the deposition of Co/Pt(111) and MOKE measurements

The CV of the Pt(111) electrode in a pure supporting electrolyte containing HCl and KCl (black curve), and with added $CoCl_2$ (red curve) are shown in **Figure S1**. By comparing the CV for specimens in electrolytes with and without $CoCl_2$, electric potential ranges that are suitable for the electrodeposition and dissolution of Co/Pt(111) were determined. The ferromagnetic layer was electrodeposited on the Pt electrode at an electric potential more negative than -700 mV for a pre-determined time, depending on the thickness of the layer. The potential range for MOKE measurements is chosen to be between -400 and -600 mV to avoid the influences of the possible deposition/dissolution of the Co deposits.

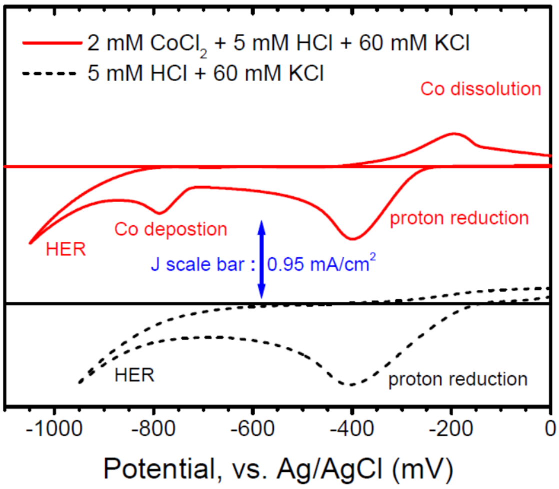

Figure S1. The CV of Pt(111) electrode in a pure supporting electrolyte containing HCl and KCl (black curve), and with a $CoCl_2$ additive (red curve).

Supplementary information 2

EDL and PZC influence the efficiency of EPTMR

The EPTMR technique is based on the characteristics of tunable H_C that is achieved by changing the electric potential in a solution process. However, the electric potential for driving the EPTMR is related to the ion species in the solution. By applying an electric potential, an electrical double layer (EDL) near the electrode surface is established.[61] The thickness of the EDL is in the nanometer scale and therefore hundreds of millivolts can create an electric field as large as 10^8 V/m.[25,62] A schematic plot in **Figure S2** shows the specimen structure where the electric field influences the ferromagnetic layer supported on the Pt electrode through the establishment of EDL. Creating a huge electric field that influences the ferromagnetic layer on the electrode can be achieved by applying electric potentials using this apparatus. The electric field is responsible for the change of MAE and related change of the H_C.

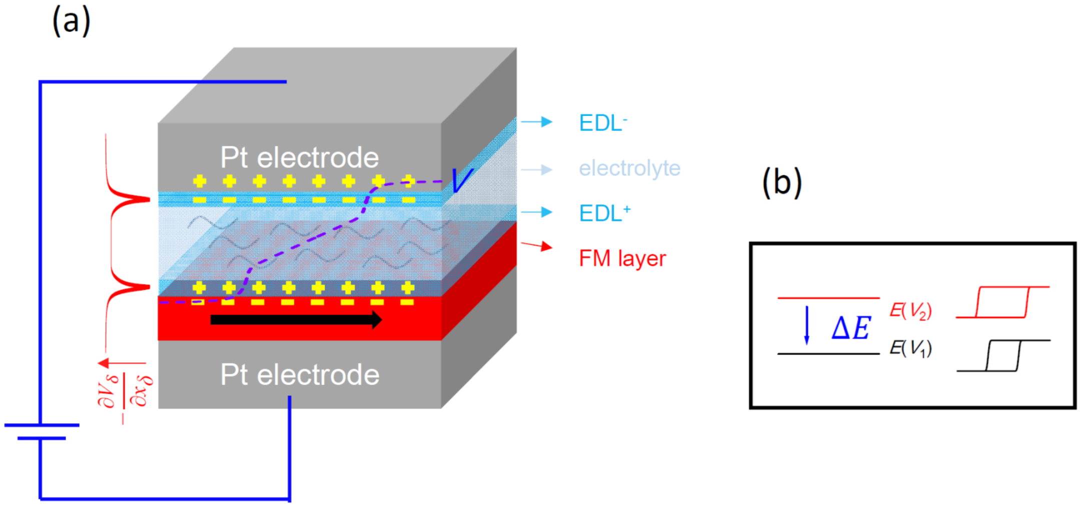

Figure S2. Schematic plots showing (a) the electrolytic cell and the huge electric field created by EDL in electrolytic environment, and (b) the electric potential induced change of the coercive force.

In different electrolytes, the surface species are different, and the effects of point of zero charge (PZC) therefore need to be taken into consideration. The PZC, related to the phenomenon of adsorption, describes the condition for which the electrical charge density on a surface is zero. For systems where H^+/OH^- are the potential-determining ions, PZC is usually determined in relation to an electrolyte's pH. In electrochemical conditions, the electrode-electrolyte interface is generally charged. If the electrode is polarizable, then its surface charge depends on the electrode potential. For a specific adsorbate δ, the effective electric potential V_δ can be expressed by

$$V_\delta = V + \Delta V_{PZC} \quad \text{(S1)};$$

where V is the applied potential on the working electrode (WE) and the ΔV_{PZC} is the potential term related to the PZC. Therefore, the relationship between the electric field and different ion species δ may be expressed as

$$E = -\frac{\partial V_\delta}{\partial x_\delta} \quad \text{(S2)}.$$

where x_δ is the thickness of EDL that depends on the ion species of the electrolyte in solution process. Based on Equations (10), (S1), and (S2), we may extend the Equation (10) in different electrolyte as

$$\Delta H_C(t_{FM}) \propto (\beta \cdot \langle -\frac{\partial (V + \Delta V_{PZC})}{\partial x_\delta} \rangle)/t_{FM}^2 \quad \text{(S3)}.$$

Based on Equations (S3), selecting suitable ion species in solution process might improve the efficiency of EPTMR technique and they may be of importance for further applications in the case of solution processes.
